# Degradation of Polylactide and Polycaprolactone as a Result of Biofilm Formation Assessed under Experimental Conditions Simulating the Oral Cavity Environment

**DOI:** 10.3390/ma15207061

**Published:** 2022-10-11

**Authors:** Dawid Łysik, Piotr Deptuła, Sylwia Chmielewska, Robert Bucki, Joanna Mystkowska

**Affiliations:** 1 Institute of Biomedical Engineering, Bialystok University of Technology, 15-351 Bialystok, Poland; 2 Department of Microbiological and Nanobiomedical Engineering, Medical University of Bialystok, 15-222 Bialystok, Poland

**Keywords:** biomaterial, polymer, microbial degradation, biofilm, artificial saliva, mucins

## Abstract

Polylactide (PLA) and polycaprolactone (PCL) are biodegradable and bioabsorbable thermoplastic polymers considered as promising materials for oral applications. However, any abiotic surface used, especially in areas naturally colonized by microorganisms, provides a favorable interface for microbial growth and biofilm development. In this study, we investigated the biofilm formation of *C. krusei* and *S. mutans* on the surface of PLA and PCL immersed in the artificial saliva. Using microscopic (AFM, CLSM) observations and spectrometric measurements, we assessed the mass and topography of biofilm that developed on PLA and PCL surfaces. Incubated up to 56 days in specially prepared saliva and microorganisms medium, solid polymer samples were examined for surface properties (wettability, roughness, elastic modulus of the surface layer), structure (molecular weight, crystallinity), and mechanical properties (hardness, tensile strength). It has been shown that biofilm, especially *S. mutans*, promotes polymer degradation. Our findings indicate the need for additional antimicrobial strategies for the effective oral applications of PLA and PCL.

## 1. Introduction

Polylactide (PLA) and polycaprolactone (PCL) are commonly used biodegradable and bioabsorbable aliphatic polyesters in a variety of biomedical applications [1,2,3,4]. Recently, PLA and PCL have been considered as promising materials in oral and pharyngeal appliances, including for 3D printed dentures [5,6], drug-enriched denture coatings [7], pharyngeal and laryngeal implants [8,9], and mucosal drug carriers [10]. Specific conditions for biofilm formation on the abiotic surfaces in the semi-open oral environment can create susceptibility to infection and reduce the reliability of polymeric medical devices. This study looks into the processes occurring on the polymer–biofilm interface.

In vivo, the degradation of PLA and PCL is dominated by non-enzymatic mechanisms, mainly hydrolysis, during which polymer absorbs water and their chains break down into smaller pieces over time [11]. This process leads to polymer erosion and alters their structure and properties, including the deterioration of mechanical strength [12]. PLA and PCL degradation kinetics depend on the polymer and environmental factors. In general, PLA degrades faster than PCL, and in the blends of these polymers, the proportions can regulate the degradation time [13]. Due to the chirality of the lactide molecule in two optically active forms (L-lactide and D-lactide), PLA can polymerize in various forms, such as poly (L-lactide) (PLLA), poly (D-lactide) (PDLA), poly (D, L-lactide) (PDLLA), and mesopolylactide. Of these, PLLA and PDLLA are mainly used for biomedical applications. Depending on the D-isomer content of PLA, fully crystalline PLLA degrades slower than amorphous PDLLA [14]. Large samples of these polymers (with dimensions of a few millimeters) decompose faster than small samples (films or microparticles) due to the autocatalytic hydrolysis inside the polymer [15]. The higher the initial degree of crystallinity and the crystallite size, the faster the degradation progresses [16]. Other material-related factors, including fillers and porosity, have been extensively discussed in the literature [17,18,19].

The three main environmental-related degradation factors of the oral cavity are temperature, pH, and the diversity of the microflora. Lyu et al. [19,20] discuss the influence of the first two on the degradation rate. Our previous in vitro studies on PLA and PCL degradation in model oral cavity conditions [20] showed a significant increase in the degradation rate of these polymers at 42 °C compared to 37 °C and no impact of pH level. We also considered the aspects of saliva and its influence on the degradation rate, pointing out its significant impact on biofilm formation [21,22,23].

The problem of material degradation in the oral cavity microbiological environment is rarely addressed, although there are many indications that the presence of biofilm may contribute to faster biomaterials degradation [24,25]. In some cases, the hydrolytic degradation of PLA and PCL can be enzymatically catalyzed with the participation of microorganisms. For PLA, these include actinomycetes, such as *Amycolatopsis* [26], *Saccharothrix* [27], *Kibdelosporangium* [28], *Actinomadura* [29], *Laceyella* [30], and *Pseudonocardia* [31] which are involved in degradation mediated by proteases; bacteria, such as *Bacillus* [32], *Geobacillus* [33], *Paenibacillus* [34], *Stenotrophomonas* [35], *Pseudomonas* [36], and the types of enzymes they produce—lipases and esterases; and fungi, such as cutinase-producing *Aspergillus* [37] and *Cryptococcus* [38], protease-producing *Tritirachium* [39], and *Trichoderma* [40], for which the type of enzyme affecting degradation has not been identified. The enzymatic degradation of PCL is involved in bacteria (mainly *Pseudomonas* and *Lactobacillus* [41,42,43,44,45]) and fungi (mainly *Aspergillus*, *Candida*, *Mucor*, *Rhizopus*, and *Thermomyces*) lipases [41,46,47,48,49,50].

We designed a set of experiments in which polylactide and polycaprolactone were incubated in the specially prepared artificial saliva in the presence of common oral microorganisms, such as *Streptococcus mutans* and *Candida krusei*. We assessed the biofilm development on these polymers’ surfaces and its influence on material properties.

## 2. Materials and Methods

### 2.1. Polymer Samples

PLA (3001D, content of D isomers ~ 1.6%, *M_n_* ~ 90,000 g/mol, NatureWorks, Minnetonka, MN, USA) and PCL granules (*M_n_* ~ 45,000 g/mol, Sigma-Aldrich, Burlington, MA, USA) were dried for approximately 3 h at 45 °C. Polymer specimens were made by injection molding on a BS60 device (Borche, Kanton, P.R.C.). After that, molded specimens with dimensions of 30 mm × 5 mm × 4 mm, were pre-conditioned in the air at room temperature for 24 h.

### 2.2. Artificial Saliva

Artificial saliva (AS) was prepared based on a Phosphate-buffered saline (PBS) in which porcine gastric mucin (M1778, Sigma-Aldrich, Saint Louis, MO, USA) at a concentration of 10 g/L and xanthan gum (G1253, Sigma-Aldrich, Saint Louis, MO, USA) at a concentration of 4 g/L were dissolved.

### 2.3. Incubation Conditions

The prepared polymer samples were incubated in the 6-well polystyrene plates in the mixture of AS and the cell culture medium (Lysogeny broth and brain heart infusion) (1:1 volume). The research used two microbial strains: the fungus *Candida krusei* ATCC 6258 and the bacteria *Streptococcus mutans* ATCC 35668 (Biomaxima, Poland). Incubation was carried out at 37 °C for 56 days. The medium was changed every 48 h (the biofilm from the surface of the samples was not removed, the samples were only rinsed in PBS). Control samples were incubated in the medium. Polymer tests were carried out after 24 and 72 h and after 28 and 56 days of incubation.

### 2.4. Biofilm Characterization on the Surface of the Polymer

The crystal violet (CV) method was used to determine the biofilm mass on the surface of polymers. In the first step, the polymers in the plates were washed with PBS to remove planktonic bacteria and 0.1% CV solution made in water was added to each well. After 15 minutes of incubation at room temperature (RT), the dye was rinsed with deionized water, and then 70% ethanol solution was added to dissolve the CV. Next, a microplate reader (Varioskan LUX, Thermo Fisher Scientific, Waltham, MA, USA) measured the absorbance at a wavelength λ = 570 nm. In each measurement cycle, 3 samples were tested (*n* = 3). The obtained test results represent mean values ± SD. Additionally, biofilm morphology was characterized by scanning laser confocal microscopy (CLSM). The samples were observed immediately after being removed from the incubation containers and rinsed with water. A laser scans of the surface topography were performed on the area of 128 × 128 micrometers with an accuracy of 0.01 micrometers on the *z*-axis.

### 2.5. AFM Biofilm Topography and Surface Layer Stiffness

Using atomic force microscopy, imaging of the biofilm structure and measurements of the surface layer stiffness was performed. Measurement needles with an elasticity constant of 0.37 N/m (AppNano NITRA-TALL-V-G) were used in the research. Both to obtain topographic images and measurements of the surface layer stiffness modulus, the Quantitative Imaging mode was used, based on measurements of probe insertion force curves (at a constant speed of 300 μm/s) at each measurement point. Topographic maps covered an area of 25 μm × 25 μm with a resolution of 128 × 128 pixels. The stiffness modulus maps covered an area of 10 μm × 10 μm with a resolution of 8 × 8 pixels. The Hertz–Sneddon model was used to calculate the elastic modulus *E* from the force curves.

### 2.6. Contact Angle

An Ossila Contact Angle Goniometer (Ossila, Sheffield, UK) equipped with a digital camera and a leveling table was used to measure the surface contact angle. On the dry surface of the polymers, with the aid of a micropipette, 25 μL of deionized water was placed and the drop image on the surface was recorded for 5 s. The image was analyzed with the included software with a drop edge detection function. In each measurement cycle, 5 polymer samples (*n* = 5) were tested. The obtained test results represent mean values ± standard deviation (SD). Statistically significant differences were determined at *p* < 0.05 (Student’s *t*-test).

### 2.7. Polymer Mass Loss

The weight loss of the incubated PLA and PCL was determined by a gravimetric method using a laboratory balance (Mettler Toledo, Columbus, OH, USA) with an accuracy of 0.01 mg. After removing from the containers, the samples were rinsed several times in water and ethanol and dried in a moisture analyzer at 37 °C for 24 h, and then the weight of the dry sample (*m_d_*) was measured. The weight loss was determined by Formula (1), in which *m*_0_ is the initial mass of the sample.

(1) $$Mass\;loss=\frac{m_{0}-m_{d}}{m_{0}}\times 100\%$$

### 2.8. Hardness and Tensile Strength

The hardness of PLA and PCL was determined using a Shore durometer (type D) (Zwick Roell, Ulm, Germany) following the ASTM D2240 standard. Each time, 5 polymer samples were tested, and 6 measurements were made for each of them. The tensile strength 𝑅_𝑚_ was determined using the Zwick/Roell Z010 testing machine (Zwick Roell, Ulm, Germany) using the ISO 527 standard. The highest force obtained during the static tensile test (*F_m_*) related to the initial cross-section of the specimen (A0) was used for the calculations. Five polymer samples were tested. The results presented in the paper represent mean values ± SD.

### 2.9. Molecular Weight of Polymers Surface Layer

The average viscosity molecular weight 𝑀𝜂 of polymers (assumed in the work as the molecular weight) was estimated based on measurements of the intrinsic viscosity [𝜂] of PLA or PCL solutions (with a concentration of 0.001 g/mL) in chloroform using a capillary viscometer with an automatic viscosity measurement system (iVisc Capillary Viscometer, Lauda-Brinkmann LP, Delran, NJ, USA; Ubbelohde capillary constant *c* = 0.003 mm^2^/s^2^). The solutions were prepared by dissolving precisely cut fragments of polymer samples (50 ± 1 μm thick) (using a rotating microtome (Struers, Ballerup, Denmark)) in chloroform. The molecular weight was determined based on the Mark–Houwink Equation (2), in which *K* and *a* are constants determined for the appropriate polymer-solvent system at a given temperature (for PLA–chloroform at 25 °C: *K* = 6.06 × 10^−2^ g/cm^3^, *a* = 0.64 [51]; for PCL–chloroform at 30 °C: *K* = 1.298 × 10^−2^ g/cm^3^, *a* = 0.828 [52]).

(2) $$\left[\eta \right]=KM_{\eta }^{a}$$

In each measurement cycle, 3 polymer solutions were tested, with each viscosity test being repeated 3 times. The results represent mean values ± SD (*n* = 9).

### 2.10. Differential Scanning Calorimetry Measurements

Polymer samples were tested by Differential Scanning Calorimetry using a DSC Discovery device (TA Instruments, New Castle, DE, USA) in a heat–cool–heat mode in the range from 0 to 200 °C for PLA and from −80 to 80 °C for PCL with 5°/min heating/cooling rate. The results analyzed in the study concern the second course of heating the sample (the first heating was carried out to remove the thermal history of the sample). As in the case of the molecular weight tests, the measurements were carried out on precisely cut fragments of polylactide and polycaprolactone samples (50 ± 1 μm thick). In each measurement cycle, 5 polymer samples (*n* = 5) were tested. On this basis, the TRIOS program obtained several data, such as glass transition temperature, cold crystallization temperature, cold crystallization enthalpy Δ*H_cc_*, melting point *T_m_*, and melting enthalpy Δ*H_m_*. Crystallinity *X_c_* was determined based on Equation (3):

(3) $$X_{c}=\frac{\Delta H_{m}-\Delta H_{cc}}{\Delta H_{m}^{100}}$$

where Δ*H_m_*^100^ is the melting enthalpy of 100% crystalline PLA/PCL (93.7 J/g for PLA and 135.3 J/g for PCL).

The obtained results of thermal properties tests show mean values ± SD. Statistically significant differences were determined at *p* < 0.05 (Student’s *t*-test).

## 3. Results

**Figure 1:**
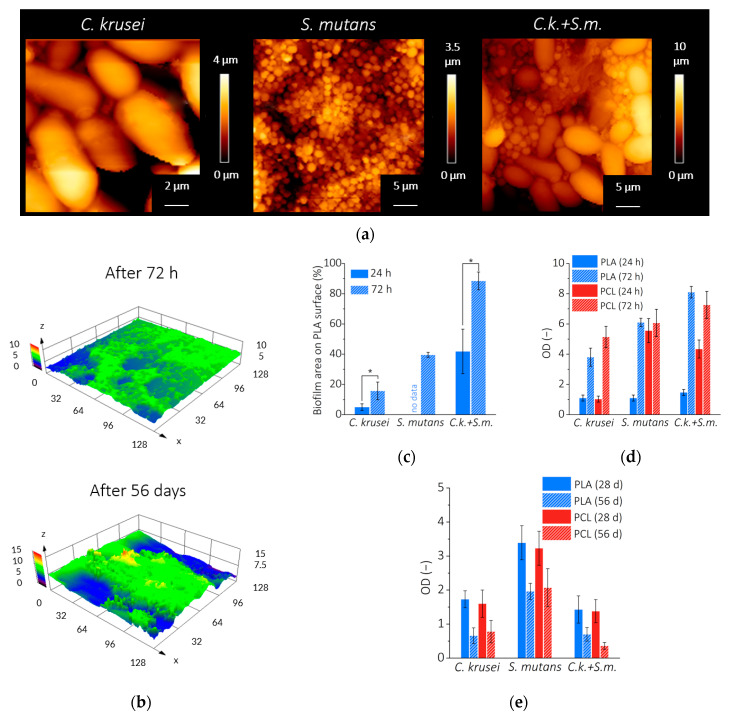
Biofilm (*C. krusei*, *S. mutans*, or *C. krusei + S. mutans*) on the surface of the tested polymers: (**a**) AFM topography of individual types of biofilm on the surface of PLA after 72 h; (**b**) CLSM topography of the *C. krusei + S. mutans* biofilm on the PLA surface after 72 h and 56 days; (**c**) biofilm development on the PLA surface during the first 72 h (based on CLSM); (**d**) biofilm development on the surface of PLA or PCL during the first 72 h (based on the optical density of the biofilm mass stained with CV); (**e**) biofilm development on the surface of PLA or PCL for 56 days (as measured by the optical density of the biofilm mass stained with CV). Results mean ± SD (*n* = 3). (*) *p* < 0.05.

The collected data describing the development of *C. krusei*, *S. mutans*, and *C. krusei + S. mutans* biofilms on the PLA and PCL surfaces are summarized in Figure 1. Figure 1a shows the AFM surface topography of biofilm on PLA after 72 h incubation. At this stage of biofilm development, individual cells can be distinguished—large and oval *C. krusei*, small and round *S. mutans*, and the structure of a mixed biofilm, where *C. krusei* cells are surrounded by *S. mutans* cells. Figure 1b shows the CLSM surface topography of *C. krusei + S. mutans* biofilm on PLA after 72 h and 56 days of incubation. The clusters of fungal cells surrounded by bacterial cells evolved into a more homogeneous structure. Figure 1c shows how the coverage of the polymer surface by the biofilm increased for the first 72 hours. After that time, *C. krusei* biofilm covered less than 20% of the surface, *S. mutans* nearly 40%, and *C. krusei + S. mutans* nearly 90%. Figure 1d shows the biofilm mass development (linearly correlated with the optical density of the CV dyed biofilm) after 24 and 72 h on the surface of PLA and PCL. The results of spectrophotometric studies are consistent with the CLSM and indicate that mixed biofilm *C. krusei + S. mutans* develops the most. After 56 days of incubation, the highest biofilm mass was observed for *S. mutans* (Figure 1e).

We believe that the biofilm on the surface of PLA or PCL may contribute to the surface layer degradation that is associated with a reduction in the physical properties of a polymer caused by changes in its chemical structure. Several tests were performed to verify this hypothesis.

**Figure 2:**
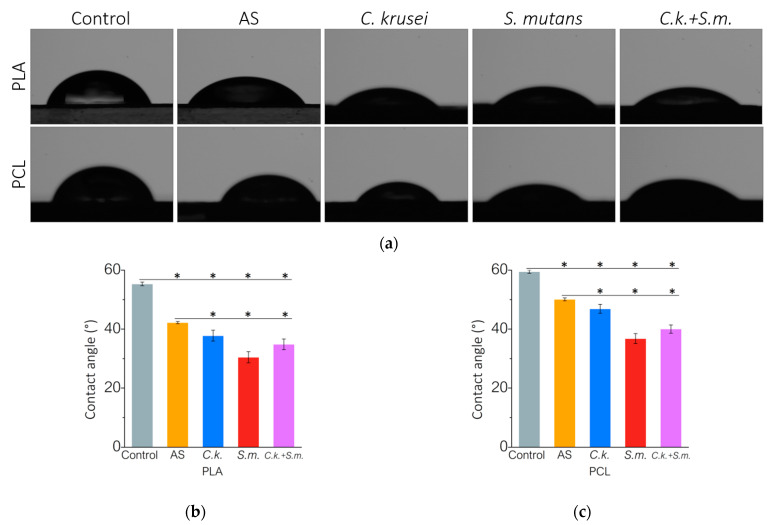
Wettability of PLA and PCL surfaces after incubation in the presence of artificial saliva and microorganisms: (**a**) water droplets on the surface of the tested polymers; (**b**) contact angle at the surface of the PLA; (**c**) contact angle at the surface of the PCL. Results mean ± SD (*n* = 3). (*) *p* < 0.05.

After removing the biofilm from the tested polymers, no visual changes were noticed between the samples incubated in artificial saliva with and without the microorganisms. However, we observe some changes in the properties of the surface layer. The results of the contact angle measurements presented in Figure 2 show that the incubation of polymers for 56 days significantly increases the hydrophilic properties of the surfaces of both PLA and PCL. In the AS, the contact angle of PLA decreased from 55° to 42°. Biofilm has intensified this phenomenon and the contact angle after 56 days dropped to 38° in *C. krusei*, to 30° in *S. mutans,* and 35° in *C. krusei + S. mutans*. A similar trend was observed for PCL, in which the contact angle after incubation in AS decreased from 59° to 50°, while in the presence of *C. krusei* microorganisms it dropped to 47°, in *S. mutans* to 37°, and in *C. krusei + S. mutans* up to 40°.

**Figure 3:**
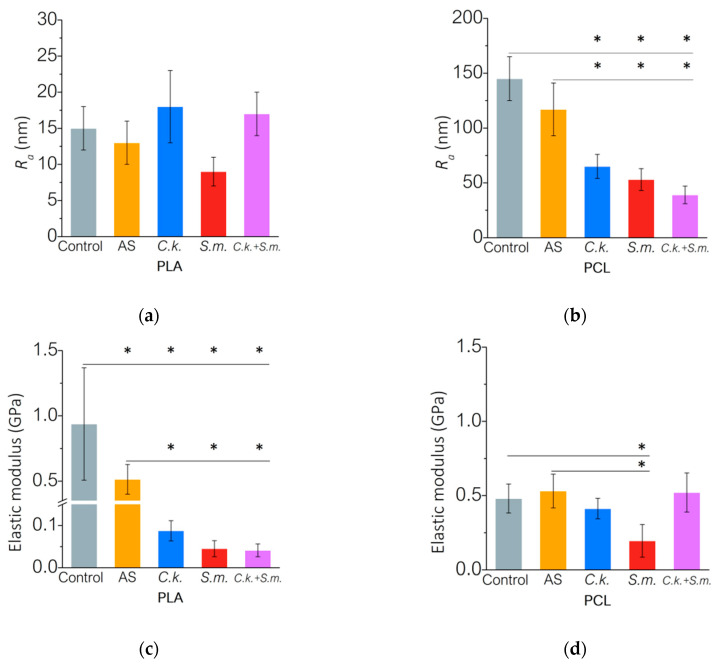
Surface properties of PLA and PCL after incubation: (**a**,**b**) roughness before and after incubation expressed by the *R_a_* parameter for PLA and PCL, respectively; (**c**,**d**) surface elasticity modulus before and after incubation for PLA and PCL, respectively. Results mean ± SD (*n* = 3). (*) *p* < 0.05.

The topography and mechanical properties of the surface layer also changed after incubation in a biological environment (Figure 3). PLA roughness measurements showed a slight increase in the *R_a_* parameter (the arithmetic average of profile height deviations from the mean line) for samples in the *C. krusei* and *C. krusei + S. mutans* environment as compared to non-incubated samples (Control) (Figure 3a). The profilometric analysis of the PCL surface showed a significant decrease in the *R_a_* value after incubation in AS and the presence of microorganisms (Figure 3b). The results of mechanical properties of the PLA surface layer show that as a result of hydrolytic degradation in AS, the elastic modulus of the surface layer decreased by nearly 45% (*E* = 514 MPa) compared to non-incubated PLA (*E* = 937 MPa). The interaction of PLA with the biological environment significantly (*p* < 0.05) changed the elastic modulus of the polymer surface layer (Figure 3c). In the presence of *C. krusei* biofilm, the elastic modulus of PLA decreased by 83% (*E* = 88 MPa) in samples incubated in AS and by over 90% in non-incubated samples. In the presence of *S. mutans* biofilm (*E* = 45 MPa) and a mixed biofilm of *C. krusei + S. mutans* (*E* = 42 MPa), the elastic modulus decreased by more than 91% compared to AS and by more than 95% compared to non-incubated samples. In the case of PCL (Figure 3d), statistically significant (*p* < 0.05) differences in the elastic modulus were observed after contact with *S. mutans* biofilm (*E* = 195 MPa), where the *E* modulus decreased by 63% compared to PCL incubated in AS (*E* = 531 MPa). It is worth emphasizing that in the mixed biofilm environment there was no decrease in the elastic modulus of the PCL surface layer as was the case with PLA.

**Figure 4:**
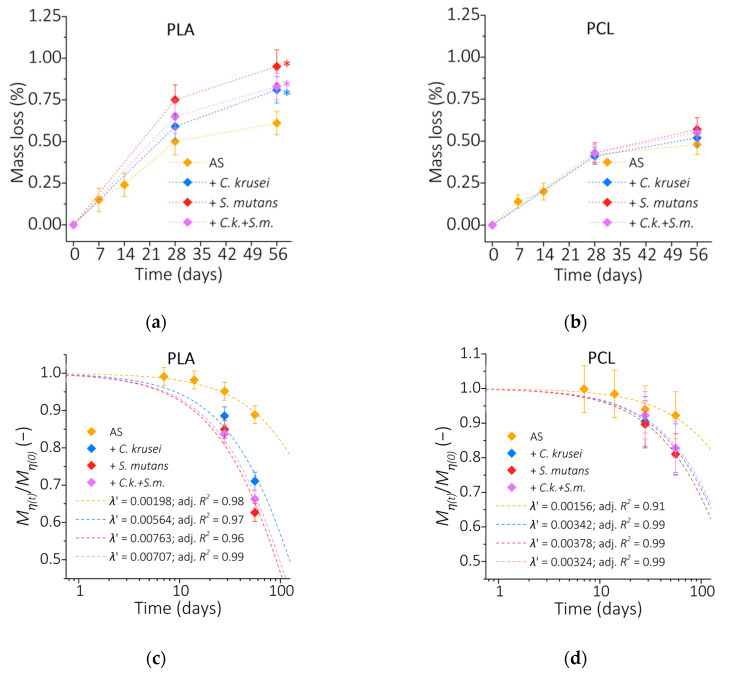
(**a**,**b**) Mass loss of PLA and PCL, respectively; (**c**,**d**) the ratio of the molecular weight after time t to the initial molecular weight of PLA and PCL, respectively. (*) *p* < 0.05.

In Figure 4a,b we show the mass loss during incubation of PLA and PCL, respectively. For PLA in the AS, after 56 days the mass loss was 0.61% but is much higher in the biofilm—in the presence of *C. krusei* the mass loss after 56 days was 0.81%, and in the presence of *S. mutans* bacteria 0.95%, and the presence of mixed biofilm 0.83%. For PCL in AS, after 56 days of incubation, the mass loss was about 0.5% and no significant changes were observed in the biofilm environment.

Polymer erosion is caused by the reduction of polymer molecular weight. In this study, we used the viscosity molecular weight *Mη* (Figure 4c,d). The initial value of *M*_*η*(*0*)_ at time “0” for PLA was 166,000 g/mol, while for PCL was 58,000 g/mol (for comparison, the values determined by the manufacturer using chromatographic methods are *Mn* ~ 90,000 g/mol for PLA and *Mn* ~ 45,000 g/mol for PCL). Changes in the molecular weight of the polymers during incubation are shown as the ratio of the molecular weight over time *M*_*η*(*t*)_ to the initial molecular weight *M*_*η*(*0*)_. The ratio *M*_*η*(*t*)_/*M*_*η*(*0*)_ decreases exponentially over time for PLA and PCL in all tested conditions, which was illustrated by fitting the exponential function *M*_*η*(*t*)_/*M*_*η*(*0*)_ = e*^-λ’t^* with the parameter *λ’* (expressed in units of 1/day) as the degradation rate (Figure 4c,d) (discussed in more detail in [12]). For PLA samples incubated in the AS, the value of *λ’* was 1.98 × 10^−3^. In the *C. krusei* biofilm, it was 5.64 × 10^−3^; in *S. mutans*, 7.63 × 10^−3^; and in the mixed biofilm, 7.07 × 10^−3^. For PCL in AS, the parameter *λ’* was 1.56 × 10^−3^; in *C. krusei*, 3.42 × 10^−3^; in *S. mutans*, 3.78 × 10^−3^; and w *C. krusei + S. mutans*, 3.24 × 10^−3^. It can be clearly stated that the presence of microorganisms on the surface of PLA during incubation in the artificial saliva significantly increases the degradation rate of the surface layer, with the highest degradation rate observed in the presence of *S. mutans*. The presence of microorganisms also increases the rate of PCL degradation.

**Figure 5:**
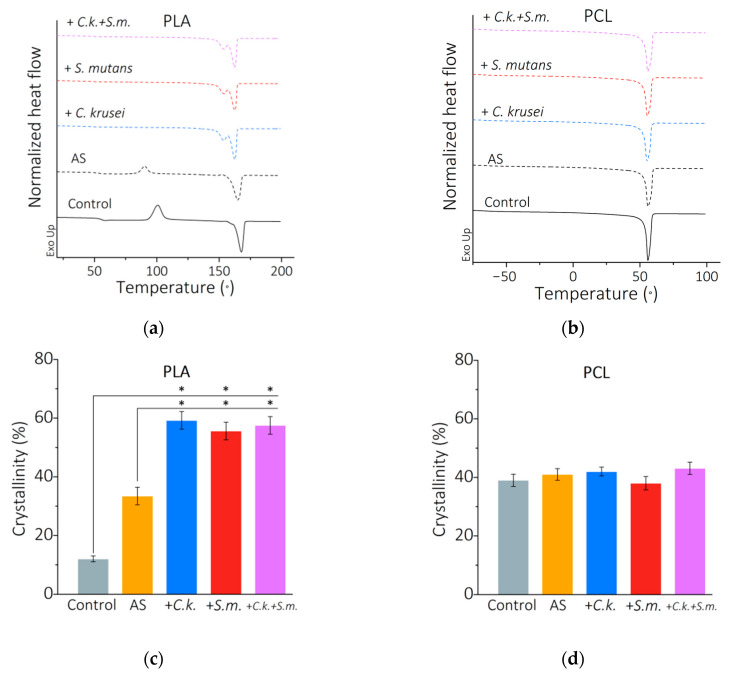
DSC of PLA and PCL after 56 days of incubation: (**a**) DSC curves (second heating) for PLA; (**b**) DSC (second heat) curves for PCL; (**c**) crystallinity for PLA; (**d**) crystallinity Xc for PCL. Results mean ± SD (*n* = 5). (*) *p* < 0.05.

The results of differential scanning calorimetry (DSC) analyses of the tested PLA and PCL polymers are presented in Figure 5. Figure 5a,b shows the DSC curves of the second heating for PLA and PCL, respectively. For the PLA control (not incubated), the glass transition temperature was ~55 °C, the cold crystallization temperature was 101 °C, the cold crystallization enthalpy was ~31 J/g, and the melting point was 168 °C, while the fusion enthalpy was ~43 J/g. After 56 days in AS, the glass transition temperature decreased to ~54 °C, the cold crystallization temperature decreased to ~91 °C, the cold crystallization enthalpy decreased to ~12 J/g, and the melting point to ~165 °C, while the melting enthalpy remained at a similar level ~43 J/g. The presence of microorganisms changed the cold crystallization enthalpy, while other properties, including the fusion enthalpy, did not change significantly. Thus, the influence of environmental conditions on the properties of polymers is most evident in the change of the crystallinity, which for PLA is the difference between the fusion enthalpy and the cold crystallization enthalpy related to the fusion enthalpy of fully crystalline PLA equal to 93.7 J/g (Figure 5c). The degree of crystallinity of non-incubated PLA was 12%, while after 56 days of incubation in AS it increased almost threefold (to ~33%). The presence of microorganisms in the artificial saliva during the incubation of PLA samples significantly (*p* < 0.05) increases their crystallinity to ~59% for *C. krusei*, ~56% for *S. mutans*, and ~57.5% for the mixed biofilm. From the DSC curves of the second PCL heating (Figure 5b), two parameters were determined—the melting point and the melting enthalpy. For the control, the melting temperature was ~56 °C and melting enthalpy ~56 J/g. Incubation in the environment of artificial saliva and microorganisms has little effect on the value of the melting enthalpy, so the changes in crystallinity are not significant (Figure 5d).

**Figure 6:**
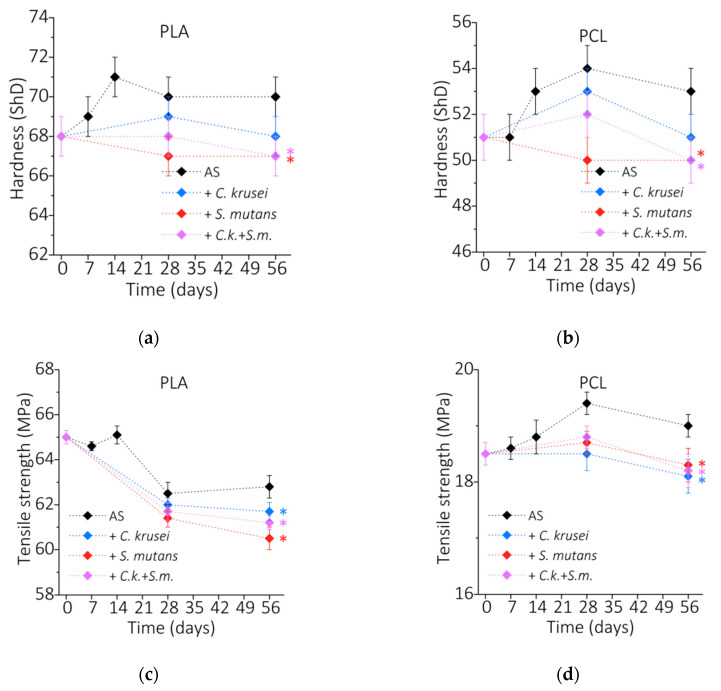
Mechanical properties of PLA and PCL during incubation in artificial saliva and microorganisms: (**a**,**b**) Shore hardness; (**c**,**d**) tensile strength. (*) *p* < 0.05.

The biofilm degradation of PLA and PCL, resulting in the erosion, decrease in molecular weight, and change of crystallinity, may cause a deterioration of polymer mechanical properties. Figure 6a shows the changes in PLA hardness during incubation. The initial PLA hardness was 68 ShD, and an increase in hardness of 71 ShD was observed during incubation in AS. After 56 days in *C. krusei,* the hardness did not change, in *S. mutans* and mixed biofilm, it dropped slightly to 67 ShD. Figure 6b shows the changes in PCL hardness during incubation. The initial hardness of PCL was 51 ShD, similar to PLA, and incubation in AS resulted in a slight increase in hardness (to 53 ShD). During incubation in the biofilm, an increase in hardness was observed after 28 days and a return to the initial value. Figure 6c shows the changes in PLA tensile strength during incubation. Incubation in AS resulted in a drop in tensile strength from 65 to about 63 MPa. The presence of microorganisms negatively affected the tensile strength, which fell below 62 MPa. Figure 6d shows the changes in the tensile strength of PCL during incubation. In AS, an initial increase in strength of about 1 MPa from 18.5 MPa was observed, followed by a decrease to 19 MPa. The presence of microorganisms did not change the strength of PCL. Overall, no significant reduction in the strength of the tested polymers was observed.

## 4. Discussion

All surfaces in the oral cavity, including implanted biomaterials, are covered with the salivary pellicle—a layer of a viscoelastic gel composed of saliva components, such as mucins MUC5B and MUC7, proline-rich proteins (gPRP, aPRP, bPRP), amylase, cystatin, statherin, histatin, and immunoglobulins (IgA, IgM). The thickness of the acquired pellicle ranges from 300 nm to even over 1000 nm [23,53]. This layer acts as an interface between the microbiological environment and tissues or biomaterials surfaces. Our previous studies [20] have shown that the incubation of polymers in artificial saliva based on porcine gastric mucins results in the adsorption of a layer with similar physicochemical properties (height, elastic modulus, pH response) to the natural acquired pellicle. This better reflects the physiological conditions in the oral cavity and allows a more accurate assessment of the processes on the biomaterial surface, especially the biofilm development and material degradation.

The processes of microbial adhesion to the acquired pellicle, the co-adhesion of microbes to microbes on the surface, and co-aggregation in the suspension are responsible for the biofilm formation in the oral cavity [54]. Intercellular interactions are specific in the oral environment and most species of microorganisms closely coexist with at least one other species. This study assessed the development of *C. krusei* and *S. mutans* biofilms as a model of bacterial and fungal strains that co-exist in the oral cavity. Both microorganisms cause oral diseases, and the development of their biofilms is a common challenge for dentists. *S. mutans* is the predominant caries pathogenic species and its main virulence characteristics are organic acid production and biofilm formation [55]. *C. krusei* belongs to the *Candida*, and together with *C. albicans*, *C. glabrata* and *C. tropicalis* are responsible for the development of oral candidiasis. It is commonly found in saliva and areas such as periodontal pockets, root canals, mucosal surfaces, and enamel as well as orthodontic appliances and dentures. *Candida* spp. actively participate in cariogenic biofilms through synergistic interaction with *S. mutans*. Their ability to secrete a large amount of extracellular matrix, combined with a large surface area of hyphal networks, supports the growth of pathogenic mixed communities. In addition, *C. krusei* has been implicated in periodontal disease, endodontic infections, and denture stomatitis [56].

In the initial stages, the mixed biofilm of both studied microorganisms developed better than single species biofilms, which indicates the possible cooperation of *C. krusei* and *S. mutans*. After 56 days of incubation, *S. mutans* biofilm developed the most. Streptococci, in particular *S. mutans*, belong to the group of early colonizers in the oral cavity and attach to the surface by recognizing molecule receptors, such as statherin, proline-rich proteins, salivary α-amylase, agglutinin, and mucins. Bamford et al. [57] showed that streptococci can increase the production of hyphae in *Candida*, which increases their adhesion to the surface and increases the survival rate of the biofilm. In later stages, the better development of *S. mutans* may be associated with the aciduricity of mature *S. mutans* biofilms, which, by secreting large amounts of organic acids, lower the pH and, due to evolutionary pressure, outcompete other microorganisms in the oral cavity [58].

The presence of mucins change the conditions for the development of such a biofilm—they reduce the virulence of pathogenic *Candida*, responsible for many types of infections [59]. The mechanism of mucin interaction takes place at the expression level of genes responsible for the development of morphological features, enabling attachment to the surface of host cells and the surface of biomaterials. Mucins also inhibit the growth of hyphae that penetrate the colonized surfaces and promote the development of biofilm [60]. The relationship of the saliva, and mucin in particular, with the microbial environment seems to be very close. The use of a saliva substitute in the biomimetics of the oral cavity, in particular the biofilm development, is a new approach to biomaterial degradation studies.

Biofilm significantly changes the surface properties of PLA and PCL, which has far-reaching consequences. The observed reduction of hydrophobicity as a result of degradation may have an impact on the further colonization of microorganisms. The general biofilm formation trends differ depending on the site (the application of the biomaterial) in the oral cavity [23]. In the subgingival areas, less biofilm is formed on hydrophobic than hydrophilic surfaces. However, such a tendency is not observed in hydrophobic supragingival areas, where the biofilm can be detached more easily by shear forces. It should be borne in mind that the presence of saliva molecules may change the surface properties of these biomaterials. Saliva molecules adsorb better to hydrophobic surfaces [61]; therefore, due to the degradation of polymeric biomaterials, the layers of mucins and other molecules may be less stable and more susceptible to shear forces. On the other hand, glycosylated mucin chain fragments may change the surface character to a more hydrophilic. However, these processes have not been fully understood so far.

In this study, the increase in the roughness of PLA samples incubated in the presence of *C. krusei* and a decrease in the presence of *S. mutans* bacteria was observed. The presence of microorganisms on the surface of PCL reduced the surface roughness. The surface topography of biomaterials is one of the key factors in microbial adhesion and biofilm development. The increase in surface roughness associated with the larger contact surface increases microbial adhesion, especially in the initial stage of biofilm development, and reduces its susceptibility to oral shear forces [62,63]. However, it should be kept in mind that the surface topography may change due to the adsorption of saliva molecules. In addition, the saliva molecules and exopolysaccharides (formed in situ by the enzymatic reactions induced by microorganisms) can locally create zones of increased adhesion and increase the virulence of bacteria, such as *S. mutans* [64].

We have also shown that the presence of microorganisms, such as *C. krusei* or *S. mutans*, reduces the elastic modulus of the surface layer both PLA and PCL. Polylactide is more susceptible to microbial degradation, in particular against *S. mutans*. The deterioration of the substrate’s mechanical properties at the nanoscale has mechanobiological consequences for further biofilm development. Studies have shown that the adhesion of microorganisms, such as *E. coli* or *Lactococcus lactis*, decreases with increasing substrate stiffness [65]. In addition, a decrease in substrate stiffness may increase the size of the microorganisms and their antibiotic susceptibility [66]. Thus, the degradation of PLA and PCL in the oral cavity may be associated with the susceptibility to biofilm development. Hypothetically, biofilm, especially fungal biofilm, may cause it to grow into the partially degraded polymer surface and cause chronic oral infections.

During 56 days of incubation, the mass loss of PLA and PCL did not exceed 1%. A greater mass loss and greater susceptibility to microbial degradation were observed for PLA. The cause of PLA erosion in the presence of *S. mutans* may be esterases and acid metabolic products, which may contribute to the catalysis of the PLA hydrolysis reaction. The erosion of PLA in the presence of esterases and other enzymes, such as lipase or proteinase K, has been investigated previously [67]. For PCL, however, no effect of these microorganisms/enzymes on weight loss was observed. Typically, the process of microbial degradation of PLA or PCL follows a similar route for most enzymes. In the first phase of microbial degradation of aliphatic polyesters, microorganisms secrete depolymerases, the production of which requires stimulation by enzymatic inducers, such as elastin, gelatine, certain proteins, or amino acids. Subsequently, the depolymerases interact with the ester bonds, resulting in the breakdown of the polymer chains into smaller segments of oligo- and monomers, which then penetrate through the cell membranes of the microorganisms into their interior, where they are broken down to carbon dioxide, water, or methane by intracellular enzymes [68].

It has been shown that the ratio of molecular weight during degradation to initial molecular weight decreases exponentially for both PLA and PCL. The shortening of polymer chains is the basic effect of degradation and affects all changes in the structure and properties of these materials. We have shown that the degradation rate of PCL is lower than that of PLA and biofilm contributes to an increase in degradation rate. These changes were much greater in PLA than in PCL. The presence of *C. krusei* biofilm nearly 3-fold increased the *λ’* value for PLA and more than 2-fold for PCL. A similar effect was observed for PCL in *S. mutans* and mixed biofilm. The presence of *S. mutans* on the surface of PLA, both in the form of a single-species biofilm and along with *C. krusei*, turned out to be a significant catalyst for the degradation processes of the surface layer, increasing the λ’ parameter over 3.5 times. So far, the processes of microbial degradation of PLA or PCL in the presence of *Candida* or *Streptococcus* has not been observed.

DSC studies show that the degradation of PLA and PCL leads to changes in the crystal structure, and the presence of biofilm on the surface accelerates these processes. This is influenced by two effects, the first one concerns the faster hydrolysis processes in amorphous rather than crystalline regions, and the second is the reorganization of the structure due to hydrolysis. In the presented studies, due to the low mass loss, the second mechanism has greater importance. Hydrolysis shortening the polymer chains facilitates the recrystallization of amorphous regions, resulting in the formation of regions with an ordered structure. When the molecular weight of the polymer drops below the solubility limit and the polymer mass begins to drop rapidly, the spaces between the chains increases, the ability to recrystallize decreases, and the first mechanism becomes more important. Degradation kinetics significantly depends on the relative amount of the amorphous phase. In subsequent degradation steps, the degree of crystallinity may decrease as the recrystallization process ceases.

Over the observed period of 56 days, for both PLA and PCL, no decrease in hardness or tensile strength was noted. This means that both the water absorption and the structural changes due to the decrease in molecular weight and the erosion of the polymer did not affect these basic properties during this period.

## 5. Conclusions

**Figure 7:**
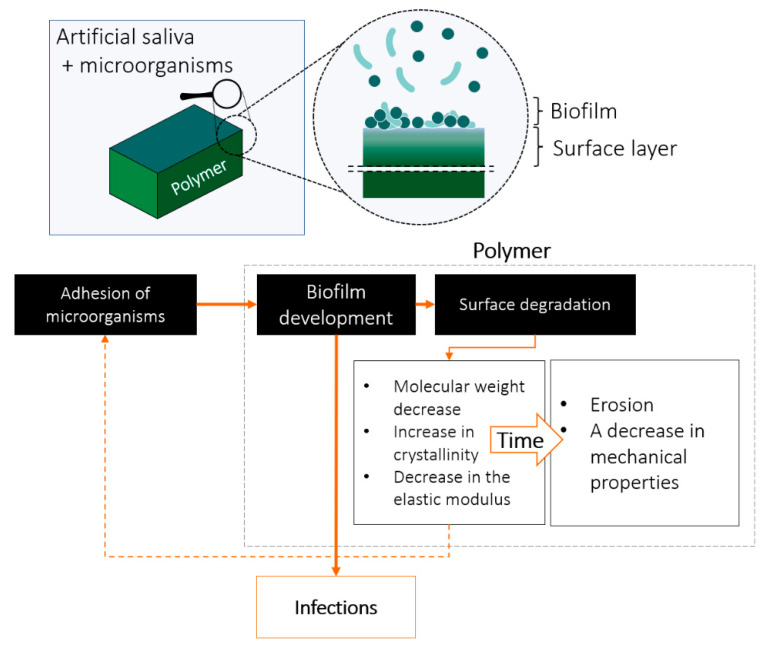
Diagram of the processes taking place at the polymer–biofilm interface in the oral cavity environment on the example of the conducted studies.

This work concerned the interaction processes at the polymer (PLA, PCL)–biofilm interface under simulated oral conditions. The key observation of the conducted research is the phenomenon of faster decomposition of the surface layer of polymers in the presence of microorganisms, which may lead to further unfavorable consequences, especially the increased colonization of microorganisms and the development of biofilm. We have summarized the processes of biofilm interaction with the surface of biodegradable polymers in Figure 7.

## Data Availability

The data presented in this study are available on request from the corresponding author.

## References

[1] Narayanan G., Vernekar V.N., Kuyinu E., Laurencin C.T. (2016). Poly (lactic acid)-based biomaterials for orthopaedic regenerative engineering. Adv. Drug Deliv. Rev..

[2] Woodruff M.A., Hutmacher D.W. (2010). The return of a forgotten polymer—Polycaprolactone in the 21st century. Prog. Polym. Sci..

[3] Santoro M., Shah S.R., Walker J.L., Mikos A.G. (2016). Poly(lactic acid) nanofibrous scaffolds for tissue engineering. Adv. Drug Deliv. Rev..

[4] James R., Manoukian O.S., Kumbar S.G. (2016). Poly(lactic acid) for delivery of bioactive macromolecules. Adv. Drug Deliv. Rev..

[5] Goyanes A., Det-Amornrat U., Wang J., Basit A.W., Gaisford S. (2016). 3D scanning and 3D printing as innovative technologies for fabricating personalized topical drug delivery systems. J. Control. Release.

[6] Norman J., Madurawe R.D., Moore C.M.V., Khan M.A., Khairuzzaman A. (2017). A new chapter in pharmaceutical manufacturing: 3D-printed drug products. Adv. Drug Deliv. Rev..

[7] Abid Z., Mosgaard M.D., Manfroni G., Petersen R.S., Nielsen L.H., Müllertz A., Boisen A., Keller S.S. (2019). Investigation of Mucoadhesion and Degradation of PCL and PLGA Microcontainers for Oral Drug Delivery. Polymers.

[8] Kang Y., Wang C., Qiao Y., Gu J., Zhang H., Peijs T., Kong J., Zhang G., Shi X. (2019). Tissue-Engineered Trachea Consisting of Electrospun Patterned sc-PLA/GO-*g*-IL Fibrous Membranes with Antibacterial Property and 3D-Printed Skeletons with Elasticity. Biomacromolecules.

[9] Gao M., Zhang H., Dong W., Bai J., Gao B., Xia D., Feng B., Chen M., He X., Yin M. (2017). Tissue-engineered trachea from a 3D-printed scaffold enhances whole-segment tracheal repair. Sci. Rep..

[10] Ensign L.M., Schneider C., Suk J.S., Cone R., Hanes J. (2012). Mucus Penetrating Nanoparticles: Biophysical Tool and Method of Drug and Gene Delivery. Adv. Mater..

[11] Pitt G.G., Gratzl M.M., Kimmel G.L., Surles J., Sohindler A. (1981). Aliphatic polyesters II. The degradation of poly (DL-lactide), poly (ε-caprolactone), and their copolymers in vivo. Biomaterials.

[12] Laycock B., Nikolić M., Colwell J.M., Gauthier E., Halley P., Bottle S., George G. (2017). Lifetime prediction of biodegradable polymers. Prog. Polym. Sci..

[13] Middleton J.C., Tipton A.J. (2000). Synthetic biodegradable polymers as orthopedic devices. Biomaterials.

[14] Tsuji H. (2005). Poly(lactide) Stereocomplexes: Formation, Structure, Properties, Degradation, and Applications. Macromol. Biosci..

[15] Grizzi I., Garreau H., Li S., Vert M. (1995). Hydrolytic degradation of devices based on poly(dl-lactic acid) size-dependence. Biomaterials.

[16] Tsuji H., Ikada Y. (2000). Properties and morphology of poly(l-lactide) 4. Effects of structural parameters on long-term hydrolysis of poly(l-lactide) in phosphate-buffered solution. Polym. Degrad. Stab..

[17] Elsawy M.A., Kim K.-H., Park J.-W., Deep A. (2017). Hydrolytic degradation of polylactic acid (PLA) and its composites. Renew. Sustain. Energy Rev..

[18] Bartnikowski M., Dargaville T.R., Ivanovski S., Hutmacher D.W. (2019). Degradation mechanisms of polycaprolactone in the context of chemistry, geometry and environment. Prog. Polym. Sci..

[19] Oksiuta Z., Jalbrzykowski M., Mystkowska J., Romanczuk E., Osiecki T. (2020). Mechanical and Thermal Properties of Polylactide (PLA) Composites Modified with Mg, Fe, and Polyethylene (PE) Additives. Polymers.

[20] Łysik D., Mystkowska J., Markiewicz G., Deptuła P., Bucki R. (2019). The Influence of Mucin-Based Artificial Saliva on Properties of Polycaprolactone and Polylactide. Polymers.

[21] Marsh P.D., Do T., Beighton D., Devine D.A. (2015). Influence of saliva on the oral microbiota. Periodontology 2000.

[22] Werlang C., Cárcarmo-Oyarce G., Ribbeck K. (2019). Engineering mucus to study and influence the microbiome. Nat. Rev. Mater..

[23] Gibbins H.L., Yakubov G.E., Proctor G.B., Wilson S., Carpenter G.H. (2014). What interactions drive the salivary mucosal pellicle formation?. Colloids Surf. B Biointerfaces.

[24] Mystkowska J., Niemirowicz-Laskowska K., Łysik D., Tokajuk G., Dąbrowski J.R., Bucki R. (2018). The Role of Oral Cavity Biofilm on Metallic Biomaterial Surface Destruction–Corrosion and Friction Aspects. Int. J. Mol. Sci..

[25] Bettencourt A.F., Neves C.B., de Almeida M.S., Pinheiro L.M., e Oliveira S.A., Lopes L.P., Castro M.F. (2010). Biodegradation of acrylic based resins: A review. Dent. Mater..

[26] Pranamuda H., Tsuchii A., Tokiwa Y. (2001). Poly (L-Lactide)-Degrading Enzyme Produced by Amycolatopsis Sp.. Macromol. Biosci..

[27] Jarerat A., Tokiwa Y. (2003). Poly(L-lactide) degradation by Saccharothrix waywayandensis. Biotechnol. Lett..

[28] Jarerat A., Tokiwa Y., Tanaka H. (2003). Poly(l-lactide) degradation by *Kibdelosporangium aridum*. Biotechnol. Lett..

[29] Sukkhum S., Tokuyama S., Kitpreechavanich V. (2009). Development of fermentation process for PLA-degrading enzyme production by a new thermophilic Actinomadura sp. T16-1. Biotechnol. Bioprocess Eng..

[30] Hanphakphoom S., Maneewong N., Sukkhum S., Tokuyama S., Kitpreechavanich V. (2014). Characterization of poly(L-lactide)-degrading enzyme produced by thermophilic filamentous bacteria Laceyella sacchari LP175. J. Gen. Appl. Microbiol..

[31] Konkit M., Jarerat A., Khanongnuch C., Lumyong S., Pathom-Aree W. (2012). Poly(Lactide) Degradation by Pseudonocardia Alni AS4.1531t. Chiang Mai J. Sci..

[32] Tomita K., Kuroki Y., Nagai K. (1999). Isolation of thermophiles degrading poly(l-lactic acid). J. Biosci. Bioeng..

[33] Tomita K., Nakajima T., Kikuchi Y., Miwa N. (2004). Degradation of poly(l-lactic acid) by a newly isolated thermophile. Polym. Degrad. Stab..

[34] Akutsu-Shigeno Y., Teeraphatpornchai T., Teamtisong K., Nomura N., Uchiyama H., Nakahara T., Nakajima-Kambe T. (2003). Cloning and Sequencing of a Poly( dl -Lactic Acid) Depolymerase Gene from *Paenibacillus amylolyticus* Strain TB-13 and Its Functional Expression in *Escherichia coli*. Appl. Environ. Microbiol..

[35] Jeon H.J., Kim M.N. (2013). Biodegradation of poly(l-lactide) (PLA) exposed to UV irradiation by a mesophilic bacterium. Int. Biodeterior. Biodegradation.

[36] Liang T.-W., Jen S.-N., Nguyen A.D., Wang S.-L. (2016). Application of Chitinous Materials in Production and Purification of a Poly(l-lactic acid) Depolymerase from Pseudomonas tamsuii TKU015. Polymers.

[37] Maeda H., Yamagata Y., Abe K., Hasegawa F., Machida M., Ishioka R., Gomi K., Nakajima T. (2005). Purification and characterization of a biodegradable plastic-degrading enzyme from Aspergillus oryzae. Appl. Microbiol. Biotechnol..

[38] Masaki K., Kamini N.R., Ikeda H., Iefuji H. (2005). Cutinase-Like Enzyme from the Yeast *Cryptococcus* sp. Strain S-2 Hydrolyzes Polylactic Acid and Other Biodegradable Plastics. Appl. Environ. Microbiol..

[39] Jarerat A., Tokiwa Y. (2001). Degradation of Poly(L-Lactide) by a Fungus. Macromol. Biosci..

[40] Lipsa R., Tudorachi N., Darie-Nita R.N., Oprică L., Vasile C., Chiriac A. (2016). Biodegradation of poly(lactic acid) and some of its based systems with Trichoderma viride. Int. J. Biol. Macromol..

[41] Gan Z., Liang Q., Zhang J., Jing X. (1997). Enzymatic degradation of poly(ε-caprolactone) film in phosphate buffer solution containing lipases. Polym. Degrad. Stab..

[42] Chen D.R., Bei J.Z., Wang S.G. (2000). Polycaprolactone microparticles and their biodegradation. Polym. Degrad. Stab..

[43] Ashton J., Mertz J., Harper J., Slepian M., Mills J., McGrath D., Geest J.V. (2011). Polymeric endoaortic paving: Mechanical, thermoforming, and degradation properties of polycaprolactone/polyurethane blends for cardiovascular applications. Acta Biomater..

[44] Castilla-Cortázar I., Más-Estellés J., Meseguer-Dueñas J.M., Ivirico J.E., Marí B., Vidaurre A. (2012). Hydrolytic and enzymatic degradation of a poly(ε-caprolactone) network. Polym. Degrad. Stab..

[45] Khan I., Dutta J.R., Ganesan R. (2017). Lactobacillus sps. lipase mediated poly (ε-caprolactone) degradation. Int. J. Biol. Macromol..

[46] Sivalingam G., Chattopadhyay S., Madras G. (2003). Enzymatic degradation of poly (ε-caprolactone), poly (vinyl acetate) and their blends by lipases. Chem. Eng. Sci..

[47] Sivalingam G., Chattopadhyay S., Madras G. (2003). Solvent effects on the lipase catalyzed biodegradation of poly (ε-caprolactone) in solution. Polym. Degrad. Stab..

[48] Ebata H., Toshima K., Matsumura S. (2000). Lipase-Catalyzed Transformation of Poly(ε-caprolactone) into Cyclic Dicaprolactone. Biomacromolecules.

[49] Yang L., Li J., Jin Y., Li M., Gu Z. (2015). In vitro enzymatic degradation of the cross-linked poly(ε-caprolactone) implants. Polym. Degrad. Stab..

[50] Sivalingam G., Vijayalakshmi S.P., Madras G. (2004). Enzymatic and Thermal Degradation of Poly(ε-caprolactone), Poly(d,l-lactide), and Their Blends. Ind. Eng. Chem. Res..

[51] Rak J., Ford J.L., Rostron C., Walters V. (1985). The preparation and characterization of poly(D,L-lactic acid) for use as a biodegradable drug carrier. Pharm. Acta Helv..

[52] Kouparitsas I.K., Mele E., Ronca S. (2019). Synthesis and Electrospinning of Polycaprolactone from an Aluminium-Based Catalyst: Influence of the Ancillary Ligand and Initiators on Catalytic Efficiency and Fibre Structure. Polymers.

[53] Amaechi B., Higham S., Edgar W., Milosevic A. (1999). Thickness of acquired salivary pellicle as a determinant of the sites of dental erosion. J. Dent. Res..

[54] Bowen W.H., Burne R.A., Wu H., Koo H. (2018). Oral Biofilms: Pathogens, Matrix, and Polymicrobial Interactions in Microenvironments. Trends Microbiol..

[55] Scharnow A.M., Solinski A.E., Wuest W.M. (2019). Targeting *S. mutans* biofilms: A perspective on preventing dental caries. MedChemComm.

[56] O’Donnell L.E., Millhouse E., Sherry L., Kean R., Malcolm J., Nile C.J., Ramage G. (2015). Polymicrobial *Candida* biofilms: Friends and foe in the oral cavity. FEMS Yeast Res..

[57] Bamford C.V., D’Mello A., Nobbs A.H., Dutton L.C., Vickerman M.M., Jenkinson H.F. (2009). *Streptococcus gordonii* Modulates *Candida albicans* Biofilm Formation through Intergeneric Communication. Infect. Immun..

[58] Matsui R., Cvitkovitch D. (2010). Acid tolerance mechanisms utilized by *Streptococcus mutans*. Futur. Microbiol..

[59] Kavanaugh N.L., Zhang A.Q., Nobile C., Johnson A.D., Ribbeck K. (2014). Mucins Suppress Virulence Traits of Candida albicans. mBio.

[60] Arevalo A.V., Nobile C.J. (2020). Interactions of microorganisms with host mucins: A focus on *Candida albicans*. FEMS Microbiol. Rev..

[61] Bansil R., Turner B.S. (2018). The biology of mucus: Composition, synthesis and organization. Adv. Drug Deliv. Rev..

[62] Anselme K., Davidson P., Popa A.M., Giazzon M., Liley M., Ploux L. (2010). The interaction of cells and bacteria with surfaces structured at the nanometre scale. Acta Biomater..

[63] Hao Y., Huang X., Zhou X., Li M., Ren B., Peng X., Cheng L. (2018). Influence of Dental Prosthesis and Restorative Materials Interface on Oral Biofilms. Int. J. Mol. Sci..

[64] Bowen W.H., Koo H. (2011). Biology of Streptococcus mutans-Derived Glucosyltransferases: Role in Extracellular Matrix Formation of Cariogenic Biofilms. Caries Res..

[65] Saha N., Monge C., Dulong V., Picart C., Glinel K. (2013). Influence of Polyelectrolyte Film Stiffness on Bacterial Growth. Biomacromolecules.

[66] Song F., Ren D. (2014). Stiffness of Cross-Linked Poly(Dimethylsiloxane) Affects Bacterial Adhesion and Antibiotic Susceptibility of Attached Cells. Langmuir.

[67] Stepczyńska M., Rytlewski P. (2018). Enzymatic degradation of flax-fibers reinforced polylactide. Int. Biodeterior. Biodegradation.

[68] Qi X., Ren Y., Wang X. (2017). New advances in the biodegradation of Poly(lactic) acid. Int. Biodeterior. Biodegradation.

